# Gene expression throughout a vertebrate's embryogenesis

**DOI:** 10.1186/1471-2164-12-132

**Published:** 2011-02-28

**Authors:** Goran Bozinovic, Tim L Sit, David E Hinton, Marjorie F Oleksiak

**Affiliations:** 1 Department of Environmental and Molecular Toxicology, Box 7633, North Carolina State University, Raleigh, NC 27695-7633, USA; 2 Department of Plant Pathology, Box 7342, North Carolina State University, Raleigh, NC 27695-7342, USA; 3 Nicholas School of the Environment, Duke University, A333 LSRC, Box 90328, Durham, NC 27708, USA; 4 Rosenstiel School of Marine and Atmospheric Sciences, University of Miami, 4600 Rickenbacker Causeway, Miami, FL 33149, USA; 5 Current Address: Division of Biological Sciences, York Hall 4070B, 9500 Gilman Drive, University of California at San Diego, La Jolla, CA 92093, USA

## Abstract

**Background:**

Describing the patterns of gene expression during embryonic development has broadened our understanding of the processes and patterns that define morphogenesis. Yet gene expression patterns have not been described throughout vertebrate embryogenesis. This study presents statistical analyses of gene expression during all 40 developmental stages in the teleost *Fundulus heteroclitus *using four biological replicates per stage.

**Results:**

Patterns of gene expression for 7,000 genes appear to be important as they recapitulate developmental timing. Among the 45% of genes with significant expression differences between pairs of temporally adjacent stages, significant differences in gene expression vary from as few as five to more than 660. Five adjacent stages have disproportionately more significant changes in gene expression (> 200 genes) relative to other stages: four to eight and eight to sixteen cell stages, onset of circulation, pre and post-hatch, and during complete yolk absorption. The fewest differences among adjacent stages occur during gastrulation. Yet, at stage 16, (pre-mid-gastrulation) the largest number of genes has peak expression. This stage has an over representation of genes in oxidative respiration and protein expression (ribosomes, translational genes and proteases). Unexpectedly, among all ribosomal genes, both strong positive and negative correlations occur. Similar correlated patterns of expression occur among all significant genes.

**Conclusions:**

These data provide statistical support for the temporal dynamics of developmental gene expression during all stages of vertebrate development.

## Background

Much effort has been expended to define developmental stages: cellular and morphological hallmarks of critical points during embryogenesis. Stages, unlike developmental time alone, provide insights into cellular and molecular processes as simple as the eight-cell stage or as complex as the onset of circulation. Although numerous aspects of development have been discovered through studies of diverse species [1-3], a comprehensive analysis of gene expression for each separate stage of vertebrate development is lacking.

Among vertebrates, developmental processes are shared [4,5]; thus insights from fish inform human studies [4,5]. *Fundulus heteroclitus*, similar to zebrafish and medaka (rice fish), has external development and transparent eggs, which facilitate associations between morphological developmental changes and patterns of gene expression. Unlike many other developmental models, *F. heteroclitus *has a ~14 day development that allows greater precision in defining specific stages than is possible in vertebrate species with short developmental times. Finally, this species has large population sizes, a well-described phylogeny, and locally adapted populations making it an exceptional model for environmental and evolutionary studies [6].

**Figure 1:**
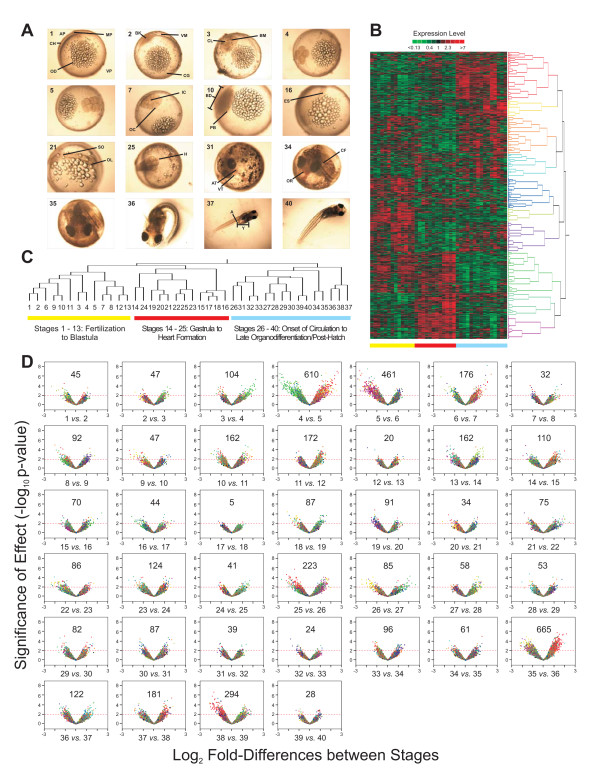
**Stages and patterns of gene expression during development**. **A**. Key stages during *F. heteroclitus *development. Stages (S) are in the upper left of each embryo image. S1, unfertilized egg. S2, 1 cell stage. S3, 2 cell stage. S4, 4 cell stage. S5, 8 cell stage. S7, 32 cell stage. S10, early blastula. S16, pre-mid gastrula. S21, 3-4 somites. S25, onset of circulation. S31 and S34, growth and organodiffertiantion. S35, pre-hatching. S36, hatching. S37, growth. S40, initiation of larval stage. (See Additional Files 1 and 4). AP-animal pole; AT-atrium; BD-blastoderm; BK-blastodisk; BM-blastomere; CF-caudal fin; CG-cortical granules; CH-chorion; CL-cleavage; ES-embryonic shield; H-heart; IC-inner cells; MP-micopyle; OC-outer cells; OD-oil droplet; OL-optic lobe; OR-oral cavity; PB-periblast; SO-somite; VM-vitelline membrane; VP-vegetal pole; VT-ventricle; Y-yolk. (For full atlas, see Additional Files 1, 2 and 4). **B**. Hierarchical clustering of 6,551 genes (95.5% of 6,857) that changed significantly between any two stages (p < 0.01). Each row represents one gene and each column represents one of forty developmental stages. Clusters of genes with similar expression patterns are shown on the right (gene tree). Red indicates high expression levels and green represents low expression levels. **C**. Hierarchical clustering of forty developmental stages based on shared gene expression patterns. Three majors clusters are colored yellow, red and blue and correspond to gene expression patterns. **D**. Pairwise differences between adjacent stages of *F. heteroclitus *development. Significances of differences as - log_10_(p-values) are plotted against log_2 _differences in expression of adjacent stages. - log_10_(p-values) range from 0 to 8 and log_2 _differences in expression range from -3 to 3 (-8-fold to 8-fold differences in expression). Numbers of significant genes (p < 0.01) that differ between stages are shown. Colors in these plots correspond to the colors of the gene tree in 1B.

To provide quantitative and statistical analyses of development, we used four biological replicates from all 40 developmental stages (from fertilization to free swimming larvae, Figure 1A; Additional File 1 shows the full *in vivo *morphological atlas and Additional File 2 shows late organodifferentiation histology) of *F. heteroclitus*. These biological replicates provide the data for statistical analyses of the expression of 6,857 genes throughout embryogenesis and a better understanding of the differences among stages and developmental pathways. Although 6,857 genes are not the full complement of genes expressed in vertebrates, they provide a statistically robust measure of differences between stages, which is important for experimental sciences that explore embryo responses to altered environments, chemical exposures and physiological differences.

## Results and Discussion

Expression of 1,607 genes (23% of 6,857) differs significantly (p < 0.01) among all stages. Our *a priori *query addressed the number of significant differences between adjacent stages: expression of 3,062 genes (45%) differs significantly between stages. Hierarchical clustering of these genes (Figure 1B and 1C) groups stages into three main clusters: 1) stages 1-13: fertilization-early gastrula; 2) stages 14-25: blastoderm-heart formation; 3) stages 26-40: onset of circulation-late organogenesis and post-hatch. These groups are in developmental order with a few exceptions (Figure 1C). Thus, the patterns of gene expression recapitulate the series of developmental stages.

The number of genes that alter expression between adjacent stages differs widely, from 5 to 665 (Figure 1D Additional File 3). At a critical p-value of 0.01, one expects approximately 70 differences due to type-1 errors. Fifteen pairs of adjacent stages have less than this false expectation. Three of these pairs of adjacent stages with few significant genes occur among gastrulation stages (12-13, 16-17, 17-18) and two are prior to the 4-cell stage (1-2, 2-3). Not surprisingly, few differences in expression occur between fertilization and first cell division. In contrast, the lack of changes in expression throughout formation of the germ layers during gastrulation is unexpected.

Expression of 610 (8.9%) and 461 (6.7%) genes are significantly different between the 4-cell (stage 4) and 8-cell (stage 5) stages and the 8-cell and 16-cell (stage 6) stages, respectively. During zebrafish embryogenesis, these stages correspond to the timing of maternal gene degradation and onset of embryo gene activity [7]. The large number of significantly differently expressed genes suggests similar timing for *F. heteroclitus*. Notice, a large number of genes show both significant increases and decreases from stage 4 to 5 (Figure 1D). However, between stages 5 and 6, most significant differences (83%) are increases in expression (negative value for log_2 _values of stage 5 - log_2 _values of stage 6) suggesting that initiation of embryonic gene expression becomes more dominant at stage 6.

The greatest number of differentially expressed genes (665, 9.7%) occurs between pre-hatching (stage 35) and hatching (stage 36), which validates our incentive to distinguish these two stages in contrast to previous work [8]. Other notable differences occur between stages 38 and 39 (294, 4.3% of genes), when most of the yolk is consumed by the free-swimming *Fundulus *larvae (eleutheroembyros [9]) and between stages 25 and 26 (223, 3.3% of genes), marked by the onset of circulation.

Times to stage have large variances (Table 1 Additional File 4 gives full stage descriptions). For example, stages 5 and 6 are on average one hour apart yet have a combined standard deviation greater than one hour. Variability in times to stage becomes more pronounced as later stages become longer (Table 1). For stages 35 and 36, with 665 differences in expression, the mean times to stage differ by fourteen hours, which is nearly equal to the standard deviation for each stage. Thus, using time alone rather than developmental markers can lead to misinterpretations of gene expression changes.

**Table 1:** Stage characterization, timing, and functional enrichment throughout

Characterization	Stage	Time (h)	Functional Enrichment	Left p-value	Right p-value	Two tail p-value
Unfertilized egg	1	0	Kinase	0.9950	**0.0187**	**0.0187**

1 cell	2	1.5 ± 0.25				

2 cells	3	2.5 ± 0.20	ATP	0.9991	**0.0065**	**0.0065**
			Fatty Acid	0.9981	**0.0122**	**0.0122**

4 cells	4	3.0 ± 0.44	Transcription Factor	**0.0309**	0.9953	0.0660

8 cells	5	5 ± 0.51				

16 cells	6	6 ± 0.50				

32 cells	7	7.5 ± 0.50	Pentose Pathway	0.9954	**0.0476**	**0.0476**

Early Morula	8	8.5 ± 0.51	Translation	0.9919	**0.0254**	**0.0254**

Late Morula	9	9.5 ± 0.51	Hatching	1	0.0017	0.0017
			Ubiquitination	0.9973	**0.0207**	**0.0207**

Early Blastula	10	10 ± 0.70	Calcium Oxidative	0.9958	**0.04832**	**0.0432**
			Phosphorylation	0.9969	**0.0114**	**0.0114**
			Post Translational	0.9994	**0.0047**	**0.0047**
			Signalling	0.9974	**0.0154**	**0.0154**

Flat Blastula	11	12 ± 1.32	Ribosomal	**0.0002**	1	**0.0002**
			Transcription Factor	**0.0034**	0.9994	**0.0061**

Pre-early Gastrula	12	15 ± 1.83				

Early Gastrula	13	19 ± 1.63				

Blastoderm	14	21 ± 1.25				

Pre-mid Gastrula	15	25 ± 2.38	DEAD	0.9999	**0.0046**	**0.0046**

Pre-mid Gastrula	16	28.5 ± 2.24	Fatty Acid	0.9976	**0.0060**	**0.0077**
			Kinase	**0.0065**	0.9984	**0.0112**
			Oxidative			
			Phosphorylation	0.9790	**0.0425**	0.0633
			Protease	0.9976	**0.0058**	**0.0067**
			Ribosomal	1	**< .0001**	**< .0001**
			Translation	0.9980	**0.0049**	**0.0077**

Mid-Gastrula	17	31 ± 1.75				

Late Gastrula	18	34 ± 1.75	Transcription Factor	0.9986	**0.0067**	**0.0067**

Early Neurula	19	38 ± 2.51	Glycolysis	0.9993	**0.0038**	**0.0038**
			Ribosomal	0.9987	**0.0042**	**0.0042**

Late Neurula	20	42 ± 3.42	Kinase	0.9954	**0.0237**	**0.0237**

3-4 Somites	21	44 ± 4.67	Transcription Factor	0.9895	**0.0475**	**0.0475**

6-9 Somites	22	49 ± 4.42	Glutathione	0.9974	**0.0318**	**0.0318**
			Oxygen	0.9988	**0.0115**	**0.0115**
			Ribosomal	0.9900	**0.0100**	**0.0100**
			Transcription Factor	**0.0231**	1	0.0535

Heart Formation	23	54 ± 5.39	ATP	0.9978	**0.0134**	**0.0134**
			Ribosomal	0.9995	**0.002**	**0.002**

Heart Beat Initiation	24	65 ± 5.32	Protease	0.9994	**0.0028**	**0.0028**
			Starch	0.9969	**0.0359**	**0.0359**
			Transcription Factor	0.9907	**0.0249**	**0.0381**

Onset of Circulation	25	72 ± 5.22	ATP	0.9991	**0.0055**	**0.0055**
			Oxygen	1	**< .0001**	**< .0001**

Growth and Organo-differentiation	26	80 ± 5.71				
	
	27	90 ± 7.80				
	
	28	102 ± 11.35	Transcription Factor	0.9860	**0.0430**	**0.0430**
	
	29	110 ± 13.31	Inositol Signalling			
			Pathway	0.9969	**0.0237**	**0.0237**
			Post-translational	0.9999	**0.0009**	**0.0009**
			Ribosomal	**0.0183**	0.9974	**0.0312**
	
	30	120 ± 13.31				
	
	31	140 ± 12.06	Ribosomal	**0.0405**	1	0.0076
			Steroid	0.9996	**0.0052**	**0.0052**
			Ubiqutination	0.9916	**0.0468**	**0.0468**
	
	32	160 ± 11.72	Pentose Pathway	0.9989	**0.0194**	**0.0194**
	
	33	180 ± 10.31	Channels	0.9975	**0.0152**	**0.0152**
			Ribosomal	**0.0044**	1	**0.0108**
			RNA	0.9921	**0.0248**	**0.0248**
	
	34	195 ± 9.46	Fatty Acid	**0.0462**	1	0.0754

Pre-hatching	35	212 ± 12.80	Ribosomal	**0.0215**	0.9951	0.0503
			Ribosomal	**< .0001**	1	**< .0001**
			RNA	**0.0463**	0.9854	0.0859
			Starch	0.9996	**0.0036**	**0.0036**

Hatching	36	226 ± 11.25	DEATH	0.9978	**0.0280**	**0.0280**

Growth	37	238 ± 10.01	ATP	0.9929	**0.0285**	**0.0285**
	
	38	256 ± 9.95	Structural	1	**< .0001**	**< .0001**
			Superoxide			
			Dismutase	0.9999	**0.0044**	**0.0044**
	
	39	256 ± 9.95				
	
	40	290-374	Ribosomal	**0.0186**	1	**0.0342**
		± 30.29	Structural	0.9965	**0.0109**	**0.0109**

*Fundulus heteroclitus *development. Bolded p-values are significant. Time values are averages ± standard deviations from 10 families of 3 embryos each.

**Figure 2:**
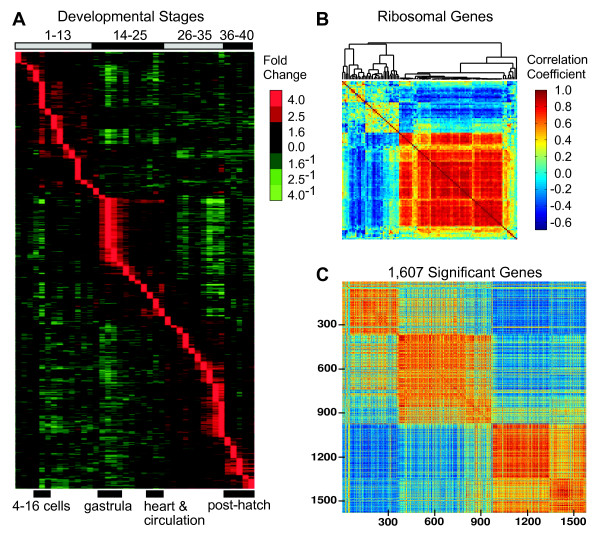
**Peak expression and correlations throughout development**. **A**. Gene expression profiles ordered by peak expression throughout development. Separate peaks were defined as within the 90% CI of the maximum transcript level measured and at least three stages away from another peak. All genes that changed significantly between any two stages (6,651 of 6,857, 97%) were ordered to gain a broad overview of gene expression throughout development. Most genes (57%) show one peak during development, 30% have two peaks, and 13% have three or more distinct peaks. 4-16 cells encompass stages 4-6. Gastrula encompasses stages 15-18. Heart and circulation encompass stages 25-23. Post-hatch encompasses stages 36-40. **B**. Correlations of ribosomal genes. Correlation coefficients > 0.4 and < -0.4 are significant at p < 0.01. Red indicates positive correlations and blue indicates negative correlations. The large group of highly positively correlated ribosomal genes has peak expression during pre-mid gastrula. **C**. Correlations among 1,607 genes significantly differently expressed across all stages (ANOVA, p < 0.01) arranged by peak order. Numbers along the left side and bottom delineate numbers of genes. Correlation coefficients > 0.4 and < -0.4 are significant at p < 0.01. Colors as in 2B.

The wave of maximum gene expression throughout development provides insight into how the magnitude of expression relates to developmental processes (Figure 2AAdditional File 3). Four distinct quadrants, similar to the hierarchical clustering results, are formed: stages 1-13 (unfertilized egg-early gastrula), stages 14-25 (blastoderm-onset of circulation), stages 26-35 (growth and organodifferentiation-pre-hatching), and stages 36-40 (hatching and growth). Pre-mid gastrulation (stage 16) has the most genes (738) with peak expression, followed by pre-hatching (stage 35, 521 genes) and the 8-cell stage (stage 5, 400 genes). Thus, although most genes have a significant increase in expression between 8 and 16 cell stages, the greatest number of genes reaches maximum expression during pre-mid gastrulation. Notably, the lack of many significant differences among gastrulation stages (15-18, Figure 1) corresponds to the large block of maximum expression. These data suggest that both qualitative differences in expression (initiation of new gene expression) and quantitative differences (as reflected in peak gene expression) are important for defining developmental processes.

Genes with peak expression during stage 16 (pre-mid gastrula) are significantly enriched for ribosomal genes (p < 2.7 × 10^-16^, Fisher exact test, Table 1), which show highly positively correlated expression patterns (Figure 2BAdditional File 5). Interestingly, these ribosomal genes are significantly negatively correlated with many of the other ribosomal genes expressed during development. Stage 16 also is significantly enriched for genes involved in oxidative phosphorylation, fatty acid metabolism, and translation as well as proteases (p < 0.0425, 0.0060, 0.0049, and 0.0067, respectively, Fisher exact tests, Table 1). Taken together, these data suggest that peak gene expression during gastrulation enhances high-energy demands during cellular proliferation and protein synthesis and turnover.

Among the 1,607 genes significantly differently expressed across all stages, almost half (49.2%) have a significant correlation coefficient (> 0.4 or < -0.4, p < 0.01): 27.5% are significantly positively correlated and 21.8% are significantly negatively correlated (Figure 2CAdditional File 6). Among pairs of genes with larger correlation coefficients, the ratio of significant positively to negatively correlated genes increases. Thus, when the absolute value of the correlation coefficients exceeds 0.5, this ratio is 1.4 (20.4%/14.4%), and when these correlation coefficients exceed 0.8, it is 6.3 (2.5%/0.4%). The numerous correlated genes suggest concerted changes in gene expression throughout development. Moreover, the increase in the relative amount of positive associations with stronger correlations suggests common regulatory factors while the less significant negative correlations may reflect coordinate regulation, potentially using similar signalling pathways, but with a variety of different regulatory factors.

**Figure 3:**
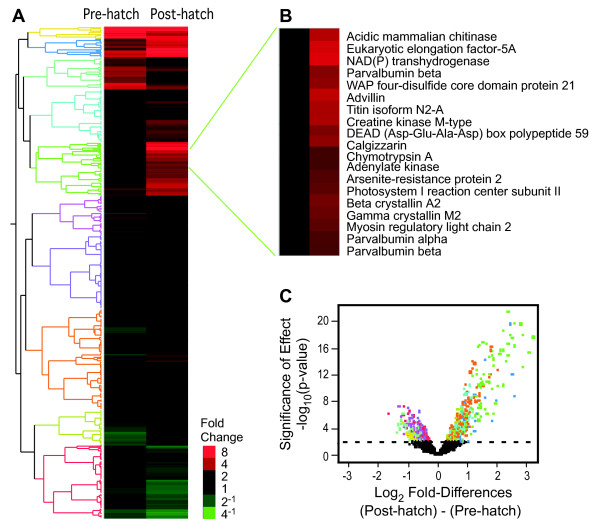
**Gene expression pre and post-hatch**. **A**. Hierarchical clustering of the 889 genes whose expression is significantly different (ANOVA, p < 0.01) between pre-hatch (stages 1-35) and post-hatch (stages 36-40) embryos. Red indicates high expression levels and green represents low expression levels. Clusters of genes with similar expression patterns are shown on the left (gene tree). The fold-change scale refers to differences in gene expression between pre- and post-hatch embryos. **B**. Enlargement of a subset of genes with high expression levels post-hatch. Thirteen unannotated genes are not included in the enlargement. **C**. Significance of pre and post-hatch expression levels. Significances of differences as - log_10_(p-values) are plotted against log_2 _differences in expression of pre and post-hatch embryos. The colors of the points correspond to the colors of the gene tree in 3A.

The last five stages (post-hatch) compared to the 35 embryonic developmental stages give insight into pre-adult *versus *developmental gene expression. Eight-hundred and eighty-nine genes (13.0%, p < 0.01) are significantly differently expressed during pre-hatching (stages 1-35) *versus *post-hatching (stages 36-40) (Figure 3); 417 genes (47%) have higher expression levels before hatching, while 472 genes (53%) are up regulated after hatching (Additional File 7). Several post-hatch up regulated genes have important functions in muscle tissue development and movement including parvalbumins alpha and beta (calcium-binding proteins involved in muscle relaxation have 5-9-fold higher expression post-hatch [10,11]), myosin regulatory light chain, skeletal muscle isoform (6.1-fold increase [12]), myosin light chain 3, skeletal muscle gene (5.2-fold increase [13]), myosin binding protein C (3.4-fold increase [14,15]) and troponins I, T and C (~3-fold increases [16]). Increased transcript levels of these genes suggest increases in movement and muscle activity of a free-swimming *Fundulus *compared to restricted movement within a chorion microenvironment before hatching. In addition, the creatine-kinase system is important for energy delivery in skeletal and cardiac muscle [17], and the 3-fold up regulation of muscle type creatine-kinase post-hatch indicates increases in metabolic activity and ATP consumption resulting from skeletal muscle activity caused by swimming.

Low transcript levels pre-hatch, and higher levels post-hatch of both retinal-cone rhodopsin-sensitve cGMP (2.4-fold increase) and photosystem I reaction center subunit II-like gene (3.6-fold increase) are associated with vision [18,19]. Since both genes are induced by light and *Fundulus *embryos are exposed to higher light intensity after hatching than within the chorion [20], the up regulation of these two genes suggest embryo photoreceptor system response to a brighter external environment.

Finally, several up regulated genes post-hatch suggest changes in metabolic activity of the free-swimming *Fundulus*. These include nucleoside diphosphate kinase NBR-B (6-fold increase), required for nucleoside triphosphate synthesis (other than ATPs) and necessary for lipid and polysaccharide synthesis, protein elongation, signal transduction and microtubule polymerization [21,22], trifunctional purine biosynthetic protein adenosine-3 (5.6-fold increase) which plays a major role in purine biosynthesis [23,24] and fatty acid-binding protein required for lipid transport and metabolism. Both liver and heart fatty acid binding protein genes are up regulated in *Fundulus *post hatch (liver: 2.3-fold, heart: 2-fold), and the increased expression levels likely reflect changes taking place within the last two stages of *Fundulus *development marked by the transition period between complete yolk consumption and increasing dependence on external food sources.

## Conclusions

Statistical analyses of nearly 7,000 *Fundulus *genes during all 40 stages of vertebrate embryogenesis highlight the temporal dynamics of developmental gene expression and stage clustering. Analyses of differences in gene expression between adjacent stages and onset of peak gene activity emphasize the importance of correctly identifying stages during embryogenesis. These data show clear differences between the transitions among stages: 4-16 cell stages have many more significant changes than similar stages (*e.g*., onset of early blastula has 1/10 the number of significant genes); pre-post hatching involves the largest number of significant temporal changes in gene expression associated with transition from egg enclosed embryos to free swimming larvae. These statistical analyses are possible because gene expression was quantified with biological replication among well-defined stages.

## Methods

### Fish field collection and maintenance

Adult *Fundulus heteroclitus *were captured from King's Creek, VA (37° 17' 54.04"N; 76° 25' 32.06"W) using minnow traps and transported under controlled temperature and aeration conditions to NCSU Aquatic Laboratory. Fish were maintained at 20°C and 15 ppt salinity in 40 gallon flow-though re-circulating tanks under a pseudo-summer light cycle (16 h light/8 h dark) for 2 months prior to embryo culturing. Effluent from the tanks was passed through an activated charcoal filter system, and 20% of the water was changed weekly. Fish were fed (brine shrimp flake, blood meal flake, and Spirulina flake - FOD, Aquatic Biosystems), and checked for health status daily.

### Fish spawning, fertilization, development, and egg collection for atlas, histology and gene expression

To minimize variability, we used embryos from a single *Fundulus *population, developing under controlled laboratory conditions. Mature females were stripped of their eggs and sperm from mature males were collected in separate beakers. Eggs from multiple females were fertilized by sperm from multiple males, and excess sperm were removed. Fertilized embryos were maintained in Petri dishes half submerged in 15 ppt filtered seawater in a 25°C environmental chamber under light during the first two stages of development (2-cell stage). Embryos that successfully reached the 2-cell stage within 3 hours were incubated under a 16 hour light/8 hour dark photoperiod at 25°C in the environmental chamber (818 Low Temperature Illuminated Incubator, Precision Scientific, USA). Fertilization success and embryo progress was monitored daily by examining representative stages during pre-determined time periods ([8]; internal data) using a dissecting stereo microscope (Nikon SME1500, Japan). Time to stage, normal *versus *abnormal development, and mortality also were recorded. Unfertilized eggs, malformed and/or dead embryos were removed from the population, and times and stages of arrest and abnormal development were recorded accordingly. Once the normally developing embryos reached pre-determined developmental stages, embryos were photographed using a Micropublisher 5.0 RTV Camera (QImaging) fitted on the stereo microscope, immediately placed in pre-chilled 1.5 ml microfuge tubes and snap-frozen at -80°C for later RNA analyses.

### Average time to stage and heart rate

To determine the average time to stage for all 40 stages, three embryos from 10 different families (each family consisted of offspring from a single female and male cross) were monitored in individual 20-ml scintillation vials. Identification of each stage was determined using a dissecting stereo microscope (Nikon SME1500, Japan) at 70-80× magnification. Multiple images of developing embryos were taken at different phases of each developmental stage. Images were captured with the Micropublisher 5.0 RTV Camera (QImaging), and catalogued, stored, and analyzed using QCapture Pro imaging software.

We calculated average times during which >50% of observed embryos showed most of the morphological characteristics of a particular stage. The embryos were observed during pre-determined time periods based on the reported *Fundulus *developmental data [8] and our preliminary results.

The same embryos used to determine average time to stage were used to determine heart rate during early organogenesis (stage 31) and pre-hatching (stage 35). A beating heart is formed, with both chambers completely differentiated and in full view by stage 31, and the heart rate can be accurately determined from that stage on. Individual embryos were placed on a depression slide under the dissecting stereo microscope for 1 minute prior to taking heart rate measurements so that the stressed embryo could re-establish resting heart beat (most *Fundulus *embryos temporarily arrest their heart beat due to a sudden change of environment, such as transfer from the petri dish to a well-lit slide surface). The heart rate of each embryo was measured by counting the number of heart beats for 30 seconds (preliminary results showed no change in the average heart beat when counts were taken at either 30 second or 1 minute intervals).

Differences among embryos were analyzed with Prism Statistical Software using one-way Analysis of Variance (1-way ANOVA, p < 0.05) for time-to-stage and heart rate differences among embryo groups (families), respectively. A pairwise t-test (p < 0.05) was used to test the differences of the means between families for both time-to-stage and heart rate.

### Histology

Decisions to refer to a structure as a specific organ or tissue were made using at least three criteria:

1. Spatial - position, relationship to other structures;

2. Temporal - time at which the structure first appears;

3. Features of its tissue and cellular components.

All of these criteria were made possible by the publications of earlier works, which have defined stages in development of various fish species [8,25-28]. Observations made with stereoscopic dissection microscopes at relatively low magnification are sufficient to provide a list of structures for early cellular stages of development. Similarly, as organ systems begin to appear, take on pigmentation and/or move, their presence provides markers for consistent recognition of specific developmental landmarks.

What has been less common is provision of detailed histological sections with sufficient resolving power to recognize organs, tissues and their component cell types. Despite the strengths of differentiating stains and greater magnification and resolution, the ability to orient sections within the changing architectural plan of a developing embryo is essential for accurate characterization to organ, tissue and cellular levels of organization. In this work, we used horizontal-longitudinal, sagittal and transverse sections. Horizontal-longitudinal sections cleave the embryo into dorsal and ventral portions. This is analogous to the frontal sections of mammalian organisms. However, since the embryo is curved over the yolk sac, a single plane of section is unlikely to be maintained through the length of the embryo. Sagittal sections cleave the embryo at the midline creating equal right and left halves of the organism. Sections to the side of the sagittal section are referred to as parasagittal. Transverse sections separate rostral from caudal portions of the embryo.

Due to the fact that the embryo and the outer surface of its associated yolk are curved, spatial relationships are difficult to define in entirety. This is a result of the embryo being inside spherical membranes and positioned flat upon a spherical surface subsequently maintained through processing by the cross-linking of proteins in the fixed embryo. Given the above, histological sections yielding true planes completely defining all of the above relationships are rare, if they exist at all. Thus, we often were faced with sections skewed to some degree, along dorsal-ventral, lateral to contralateral, lateral to medial, and/or horizontal to longitudinal orientations. For continuity and for overall representation, we regard relationships seen nearly in their entirety at lower magnification (10× objective) as valuable and these are followed by analysis of smaller areas using higher magnification and for the most part, differentiating power (*i.e*., the ability to distinguish one structure from its neighbors) giving us the potential to label organs, tissues and cells.

We chose late organogenesis of *Fundulus *development as a representative stage of major histological structures. During this stage, the heart chambers are fully differentiated and all the major organ systems are developed and fully functional. Upon confirming the stage and measuring the heart rate, the embryo pictures were taken and catalogued, the embryos were fixed in 10% neutral buffered formalin for 24-48 hours and stored overnight in 30% sucrose. The embryos were punctured through the chorion once, using the tip of a hypodermic needle, transferred to the mesh tissue cassettes, and allowed to fix longer overnight due to the thickness of chorionic membranes.

Embryos were embedded in paraffin, trimmed into 100 micron blocks and reinfiltrated in paraffin, and then reimbedded into the block. Tissues were then embedded for sectioning, which was done at 5 microns and placed on Silanized coated slides. Embryos were stained with hemotoxylin and eosin. Histological sections were viewed under a Nikon Eclipse E600 microscope, and the images were taken using Lumenera Infinity 2 (model #2-2C) 2.0 megapixel, 12 fps, CCC color camera. Digital images were analyzed using Eclipse Net Version 1.16.5 software.

### Embryo RNA isolation, amplification, and labeling

Pools of frozen embryos collected at each developmental stage were used for RNA isolation, labeling, and microarray hybridization. Four pools of 25 embryos were used for stages 1-10, four pools of 15 embryos were used for stages 11-15, and 4 pools of 10 embryos were used for stages 16-40. Embryo RNA was extracted using a TRIzol^® ^buffer (Invitrogen, Carlsbad, CA, USA). Purified RNA was quantified with a spectrophotometer, and RNA quality was assessed by gel electrophoresis. RNA for hybridization was prepared by one round of amplification (aRNA) using the Amino Allyl MessageAmp aRNA Kit (Ambion, Austin, TX, USA) to form copy template RNA by T7 amplification. Amino-allyl UTP was incorporated into targets during T7 transcription, and resulting amino-allyl aRNA was coupled to Cy3 and Cy5 dyes (GE Healthcare, Piscataway, NJ, USA).

Labeled aRNA samples (2 pmol dye/ul) were hybridized to slides in 10 ul of hybridization buffer [50% formamide buffer, 5× SSPE, 1% sodium dodecyl sulfate, 0.2 mg/ml bovine serum albumin, 1 mg/ml denatured salmon sperm DNA (Sigma), and 1 mg/ml RNAse free poly(A) RNA (Sigma)] for 44 hours at 42°C. Slides were prepared for hybridization by blocking in 5% ethanoloamine, 100 mM Tris pH 7.8, and 0.1% SDS added just before use for 30 minutes at room temperature, washed for one hour in 4× SSC, 0.1% SDS at 50°C, and then boiled for 2 minutes in distilled water to denature the cDNAs. Resulting 16 bit Tiff Images were quantified using ImaGene^® ^(Biodiscovery, Inc.) spotfinding software. Controls and any gene that did not have at least one individual with a signal greater than the average signal from all herring sperm control spots (non-specific hybridization signal) plus one standard deviation were removed prior to statistical analyses. In total, 6,789 genes were analyzed.

### Microarrays

Amplified cDNA sequences for 7,000 genes from *F. heteroclitus *cDNA libraries were spotted onto epoxide slides (Corning Inc., Corning, NY, USA) using an inkjet printer (Aj100, ArrayJet, Scotland, UK). The cDNAs used for the arrays were derived from libraries made from all 40 stages of *Fundulus *development, immediately post-hatch whole larvae, and adult tissues. Each slide contained four spatially separated arrays of ~7,000 spots (genes) including controls. All spotted genes were sequenced and represent all of the unique contigs [29] isolated from the cDNA libraries. Thus, even if multiple sequences were annotated identically, they were treated as different genes. Multiple sequences with the same annotation do not contig together because: 1) they really are the same gene, but the sequences do not overlap, 2) they represent duplicate genes with different chromosomal locations, or 3) they share a high similarity (and hence are named based on this similarity) but are not the same gene. We erred on the side of caution and treated every gene-spot as unique. Each spot was analyzed as a separate gene for all analyses except the ribosomal correlations. For ribosomal correlations, only ribosomal genes that also had unique names were correlated to minimize correlations among potentially the same gene or recent gene duplicates. These ribosomal genes were chosen arbitrarily as the first gene in the gene list with a unique name.

### Experimental design for microarrays

A double loop design was used for the microarray hybridizations where each sample is hybridized to 2 arrays using both Cy3 and Cy5 labelled fluorophores [30,31]. The loop consisted of Cy3 and Cy5 labelled embryo aRNAs from 4 biological pools for each of 40 stages (S). In total, 160 biological pools were hybridized to 80 microarrays. Each array had different combinations of biological pools [32]. The double loop formed was S1 → S2 → S3→ ... S40→ S1 and S40→ S39 → S38 → ... S2 → S1 → S40 where each arrow represents a separate hybridization (array) with the biological pool at the base of the arrow labeled with Cy3 and the biological pool at the head of the arrow labelled with Cy5.

To control for batch effects, the biological replicates for each stage were randomized on arrays and were not processed simultaneously, the same batch of arrays was used for all hybridizations, and all arrays were processed within three days.

### Embryonic gene expression

Log_2 _measures of gene expression were normalized using a linear mixed model in JMP Genomics 3.2 (SAS, Cary, NC, USA) to remove the effects of dye (fixed effect) and array (random effect) following a joint regional and spatial Lowess transformation in MAANOVA version 0.98.8 for R to account for both intensity and spatial bias (Additional File 8 shows representative MA plots after normalization) [33].

The linear mixed model was of the form y_ij _= μ + A_i _+ D_j _+ (AxD)_ij _+ ε_ij _where, y_ij _is the signal from the i^th ^array with dye j, μ is the sample mean, A_i _and D_j _are the overall variation in arrays (arrays 1-80) and dyes (Cy3 and Cy5), (AxD)_ij _is the array × dye interaction and ε _ij _is the stochastic error [34,35].

Residuals from the above model were used in a linear mixed model to test for differences between stages on a gene-by-gene basis. The model was r_ijk _= μ + A_i _+ D_j _+ T_k _+ ε _ijk _where *T_k _*is the *k*^th ^treatment (stage 1-40, 39 d.f.), the D_j _effect is fixed (1 d.f.) and the A_i _effect is random (79 d.f., leaving 40 d.f. for the residual error). We used a similar analysis to test for differences between pre and post-hatch embryos except the *k*^th ^treatment represented pre-hatch (stages 1-35) and post-hatch (stages 36-40). In this analysis, we had 1 d.f. for dyes, 79 d.f. for arrays, and 1 d.f. for treatment, leaving 78 d.f. for the residual error.

For all mixed model analyses, we used a nominal p-value cut-off for significant genes of p < 0.01. Using this p-value reveals more genes that may be differentially expressed but risks identifying genes that may be false positives. Microarray data have been deposited in NCBI's Gene Expression Omnibus [36] and are accessible through GEO Series accession number GSE21372 http://www.ncbi.nlm.nih.gov/geo/query/acc.cgi?acc=GSE21372.

Hierarchical clustering used JMP Genomics 3.2, Cluster 3.0 for Mac OS X, and Java TreeView version 1.0.8 [37]. Correlation analyses were done in JMP Genomics 3.2 and MATLAB version 7.2 was used for visualization. For peak expression, genes were ordered by their time of peak expression and standardized least square means were visualized. Separate peaks were defined as within the 90% CI of the maximum transcript level measured and at least 3 stages away from another peak.

## Authors' contributions

GB and MFO designed the experiment. GB and TLS isolated and labeled embryo RNAs. GB and DEH performed morphological and histological analyses. GB and MFO performed hybridizations and statistical analyses of gene expression data and drafted the manuscript. All authors critically revised the manuscript and gave approval of the final version.

## Supplementary Material

Additional file 1 **Stages (1-40) of normal development of *Fundulus heteroclitus***. Click here for file

Additional file 2 ***Fundulus heteroclitus *embryo histology at stage 31**. Click here for file

Additional file. 3 **Lsmeans and standardized lsmeans for genes significantly differently expressed between stages**. Click here for file

Additional file 4 ***F. heteroclitus *stage descriptions**. Click here for file

Additional file 5 **Ribosomal correlations**. Click here for file

Additional file 6 **Significant gene correlations**. Click here for file

Additional file 7 **Genes significantly differently expressed between pre-hatch and post-hatch**. Click here for file

Additional file 8 **Representative pre- and post-normalization MA plots**. Click here for file

## References

[B1] Arbeitman MN Furlong EE Imam F Johnson E Null BH Baker BS Krasnow MA Scott MP Davis RW White KP Gene expression during the life cycle of Drosophila melanogaster Science 2002 297 5590 2270 2275 10.1126/science.1072152 12351791

[B2] Martindale MQ The evolution of metazoan axial properties Nat Rev Genet 2005 6 12 917 927 10.1038/nrg1725 16341072

[B3] Whitfield CW Cziko AM Robinson GE Gene expression profiles in the brain predict behavior in individual honey bees Science 2003 302 5643 296 299 10.1126/science.1086807 14551438

[B4] Zon LI Zebrafish: a new model for human disease Genome Res 1999 9 2 99 100 10022974

[B5] Ton C Hwang DM Dempsey AA Tang HC Yoon J Lim M Mably JD Fishman MC Liew CC Identification, characterization, and mapping of expressed sequence tags from an embryonic zebrafish heart cDNA library Genome Res 2000 10 12 1915 1927 10.1101/gr.10.12.1915 11116087 PMC313056

[B6] Burnett KG Bain LJ Baldwin WS Callard GV Cohen S Di Giulio RT Evans DH Gomez-Chiarri M Hahn ME Hoover CA Fundulus as the Premier Teleost Model in Environmental Biology: Opportunities for New Insights Using Genomics Comp Biochem Physiol Part D Genomics Proteomics 2007 2 4 257 286 10.1016/j.cbd.2007.09.001 18071578 PMC2128618

[B7] Mathavan S Lee SG Mak A Miller LD Murthy KR Govindarajan KR Tong Y Wu YL Lam SH Yang H Transcriptome analysis of zebrafish embryogenesis using microarrays PLoS Genet 2005 1 2 260 276 10.1371/journal.pgen.0010029 16132083 PMC1193535

[B8] Armstrong PB Child JS Stages of normal development of Fundulus heteroclitus Biological Bulletin 1965 128 2 143 168 10.2307/1539545

[B9] Balon EK Terminology of intervals in fish development J Fish Res Board Can 1975 32 1663 1670

[B10] Lannergren J Elzinga G Stienen GJ Force relaxation, labile heat and parvalbumin content of skeletal muscle fibres of Xenopus laevis J Physiol 1993 463 123 140 8246178 10.1113/jphysiol.1993.sp019587 PMC1175336

[B11] Pauls TL Cox JA Berchtold MW The Ca2+(-)binding proteins parvalbumin and oncomodulin and their genes: new structural and functional findings Biochim Biophys Acta 1996 1306 1 39 54 8611623 10.1016/0167-4781(95)00221-9

[B12] Kabaeva ZT Perrot A Wolter B Dietz R Cardim N Correia JM Schulte HD Aldashev AA Mirrakhimov MM Osterziel KJ Systematic analysis of the regulatory and essential myosin light chain genes: genetic variants and mutations in hypertrophic cardiomyopathy Eur J Hum Genet 2002 10 11 741 748 10.1038/sj.ejhg.5200872 12404107

[B13] Sachdev S Raychowdhury MK Sarkar S Human fast skeletal myosin light chain 2 cDNA: isolation, tissue specific expression of the single copy gene, comparative sequence analysis of isoforms and evolutionary relationships DNA Seq 2003 14 5 339 350 14756420 10.1080/1042517031000154952

[B14] Yasuda M Koshida S Sato N Obinata T Complete primary structure of chicken cardiac C-protein (MyBP-C) and its expression in developing striated muscles J Mol Cell Cardiol 1995 27 10 2275 2286 10.1016/S0022-2828(95)91731-4 8576942

[B15] Mohamed AS Dignam JD Schlender KK Cardiac myosin-binding protein C (MyBP-C): identification of protein kinase A and protein kinase C phosphorylation sites Arch Biochem Biophys 1998 358 2 313 319 10.1006/abbi.1998.0857 9784245

[B16] Gomes AV Potter JD Szczesna-Cordary D The role of troponins in muscle contraction IUBMB Life 2002 54 6 323 333 10.1080/15216540216037 12665242

[B17] Momken I Lechene P Koulmann N Fortin D Mateo P Doan BT Hoerter J Bigard X Veksler V Ventura-Clapier R Impaired voluntary running capacity of creatine kinase-deficient mice J Physiol 2005 565 Pt 3 951 964 10.1113/jphysiol.2005.086397 15831533 PMC1464549

[B18] Fain GL Matthews HR Cornwall MC Koutalos Y Adaptation in vertebrate photoreceptors Physiol Rev 2001 81 1 117 151 11152756 10.1152/physrev.2001.81.1.117

[B19] Nelson N Ben-Shem A Photosystem I reaction center: past and future Photosynth Res 2002 73 1-3 193 206 10.1023/A:1020403231100 16245122

[B20] Iconomidou VA Chryssikos DG Gionis V Pavlidis MA Paipetis A Hamodrakas SJ Secondary structure of chorion proteins of the teleostean fish Dentex dentex by ATR FT-IR and FT-Raman spectroscopy J Struct Biol 2000 132 2 112 122 10.1006/jsbi.2000.4307 11162733

[B21] Berg JM Tymoczko L Stryer L Biochemistry 2002 5 WH Freeman and Company

[B22] Gilles AM Presecan E Vonica A Lascu I Nucleoside diphosphate kinase from human erythrocytes. Structural characterization of the two polypeptide chains responsible for heterogeneity of the hexameric enzyme J Biol Chem 1991 266 14 8784 8789 1851158

[B23] Schild D Brake AJ Kiefer MC Young D Barr PJ Cloning of three human multifunctional de novo purine biosynthetic genes by functional complementation of yeast mutations Proc Natl Acad Sci USA 1990 87 8 2916 2920 10.1073/pnas.87.8.2916 2183217 PMC53804

[B24] Patterson D Graw S Jones C Demonstration, by somatic cell genetics, of coordinate regulation of genes for two enzymes of purine synthesis assigned to human chromosome 21 Proc Natl Acad Sci USA 1981 78 1 405 409 10.1073/pnas.78.1.405 6941256 PMC319062

[B25] Gonzales-Doncel M Okihiro MS Villalobos SA Hinton DE Tarazona JV A quick reference guide to the normal development of Oryzias latipes (Teleostei, Adrianichthyidae) Journal of Applied Ichthyology 2003 21 39 52 10.1111/j.1439-0426.2004.00615.x

[B26] Iwamatsu T Stages of normal development in the medaka Oryzias latipes Mech Dev 2004 121 7-8 605 618 10.1016/j.mod.2004.03.012 15210170

[B27] Kimmel CB Ballard WW Kimmel SR Ullmann B Schilling TF Stages of embryonic development of the zebrafish Dev Dyn 1995 203 3 253 310 10.1002/aja.1002030302 8589427

[B28] Oppenheimer JM The normal stages of Fundulus heteroclitus Anatomical Record 1937 68 1 15 10.1002/ar.1090680102

[B29] Paschall JE Oleksiak MF VanWye JD Roach JL Whitehead JA Wyckoff GJ Kolell KJ Crawford DL FunnyBase: a systems level functional annotation of Fundulus ESTs for the analysis of gene expression BMC Genomics 2004 5 1 96 10.1186/1471-2164-5-96 15610557 PMC544896

[B30] Kerr K Churchill G Experimental design for gene expression analysis Biostatistics 2001 2 183 201 10.1093/biostatistics/2.2.183 12933549

[B31] Kerr M Churchill G Experimental design for gene expression microarrays Biostatistics 2001 2 2 183 201 10.1093/biostatistics/2.2.183 12933549

[B32] Altman NS Hua J Extending the loop design for two-channel microarray experiments Genet Res 2006 88 3 153 163 10.1017/S0016672307008476 17371610

[B33] Wu H Kerr K Cui X Churchill G ″MAANOVA: a software package for the analysis of spotted cDNA microarray experiments". The Analysis of Gene Expression Data: Methods and Software 2003

[B34] Jin W Riley RM Wolfinger RD White KP Passador-Gurgel G Gibson G The contributions of sex, genotype and age to transcriptional variance in Drosophila melanogaster Nat Genet 2001 29 4 389 395 10.1038/ng766 11726925

[B35] Wolfinger RD Gibson G Wolfinger ED Bennett L Hamadeh H Bushel P Afshari C Paules RS Assessing gene significance from cDNA microarray expression data via mixed models J Comput Biol 2001 8 6 625 637 10.1089/106652701753307520 11747616

[B36] Edgar R Domrachev M Lash AE Gene Expression Omnibus: NCBI gene expression and hybridization array data repository Nucleic Acids Res 2002 30 1 207 210 10.1093/nar/30.1.207 11752295 PMC99122

[B37] de Hoon MJ Imoto S Nolan J Miyano S Open source clustering software Bioinformatics 2004 20 9 1453 1454 10.1093/bioinformatics/bth078 14871861

