# Supplementary material for: Gene expression throughout a vertebrate's embryogenesis
Source: BMC Genomics. 2011 Feb 28;12:132. doi: 10.1186/1471-2164-12-132 (PMC3062618; doi:10.1186/1471-2164-12-132)
Supplement: Additional file 1 — Stages (1-40) of normal development of Fundulus heteroclitus. [file 1471-2164-12-132-S1.PDF]

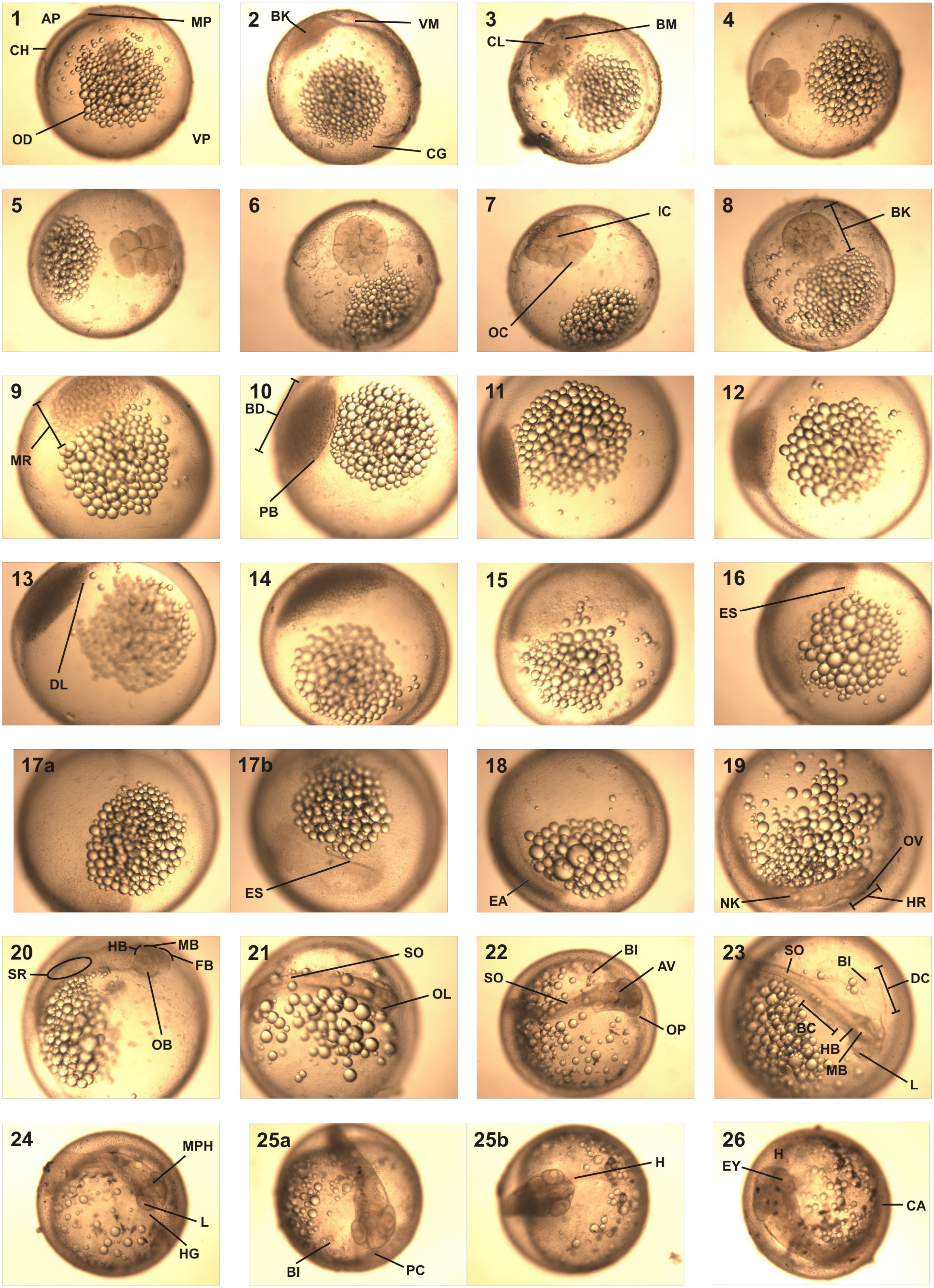
1
AP
MP
CH
OD
VP
2
BK
VM
CG
3
BM
CL
4
5
6
7
IC
OC
8
BK
9
MR
10
BD
PB
11
12
13
DL
14
15
16
ES
17a
17b
ES
18
EA
19
OV
NK
HR
20
HB
MB
SR
FB
OB
21
SO
OL
22
BI
AV
SO
OP
23
SO
BI
DC
BC
HB
MB
L
24
MPH
L
HG
25a
BI
PC
25b
H
26
H
EY
CA

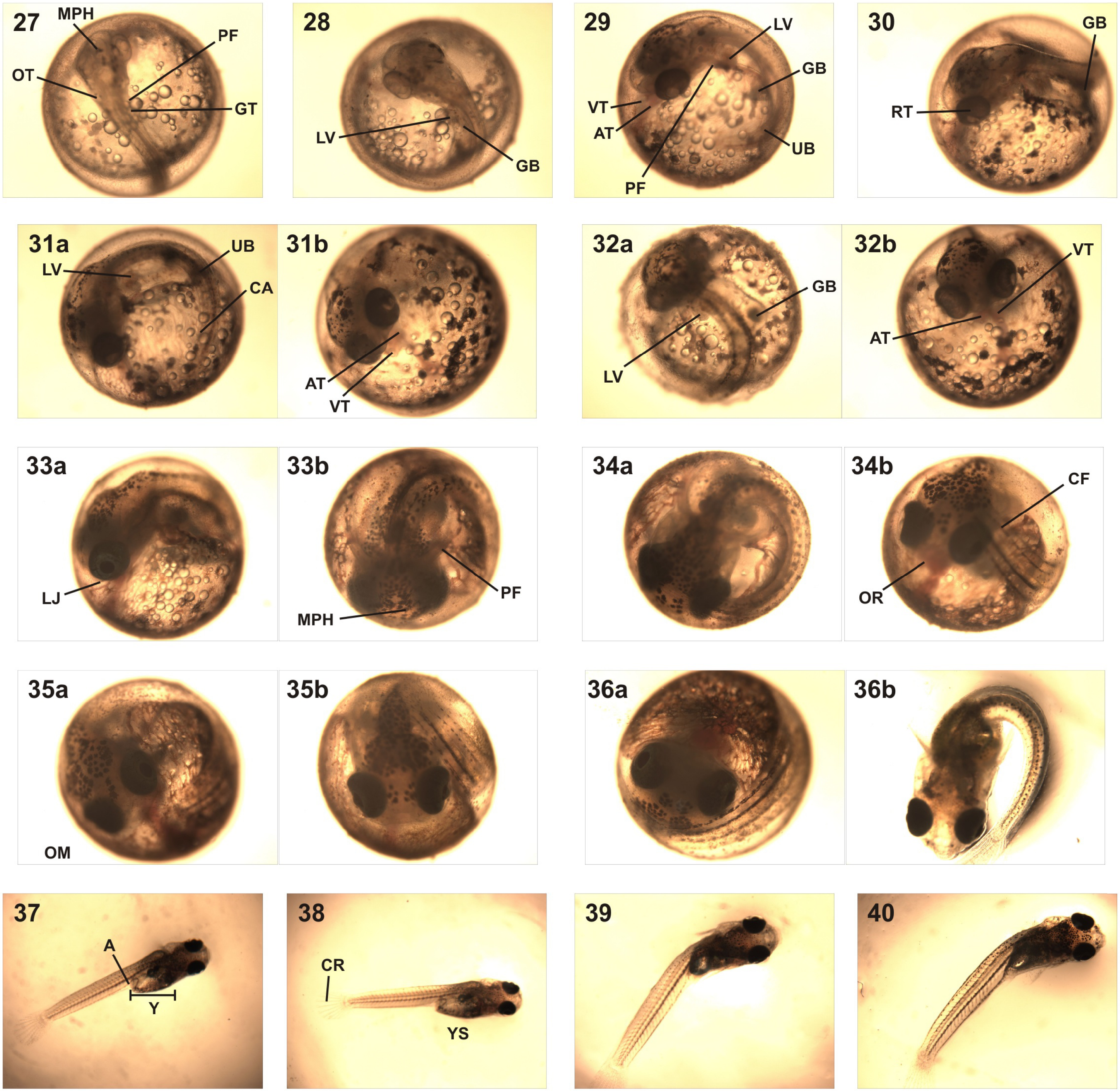

Additional File 1. Stages (1-40) of normal development of *Fundulus heteroclitus*. A-anus; AP-animal pole; AT-atrium; AV-auditory vesicle; BC-body cavity; BD-blastoderm; BI-blood islands; BK-blastodisc; BM-blastomere; CA-caudal artery; CF-caudal fin; CG-cortical granules; CH-chorion; CL-cleavage; CR-caudal fin rays; DC-duct of Cuvier; DL-dorsal lip; EA-embryonic axis; ES-embryonic shield; EY-eye; FB-forebrain; GB-gall bladder; GT-gut; H-heart; HB-hindbrain; HG-heart region; HR-head region; IC-inner cells; L-optic lens; LJ-lower jaw; LV-liver; MB-midbrain; MP-micopyle; MPH-melanophores; MR-morula; NK-neural keel; OB-optic bud; OC-outer cells; OD-oil droplet; OF-olfactory pits; OL-optic lobe; OM-operculum; OP-optic cup; OR-oral cavity; OT-otolith; OV-optic vesicle; PB-periblast; PC-pericardial cavity; PF-pectoral fin; RT-retina; SO-somite; SR-somite region; UB-urinary bladder; VM-vitaline membrane; VP-vegetal pole; VT-ventricle; Y-yolk. See Additional File 4 for stage descriptions.
