# Supplementary material for: Gene expression throughout a vertebrate's embryogenesis
Source: BMC Genomics. 2011 Feb 28;12:132. doi: 10.1186/1471-2164-12-132 (PMC3062618; doi:10.1186/1471-2164-12-132)
Supplement: Additional file 2 — Fundulus heteroclitus embryo histology at stage 31. [file 1471-2164-12-132-S2.PDF]

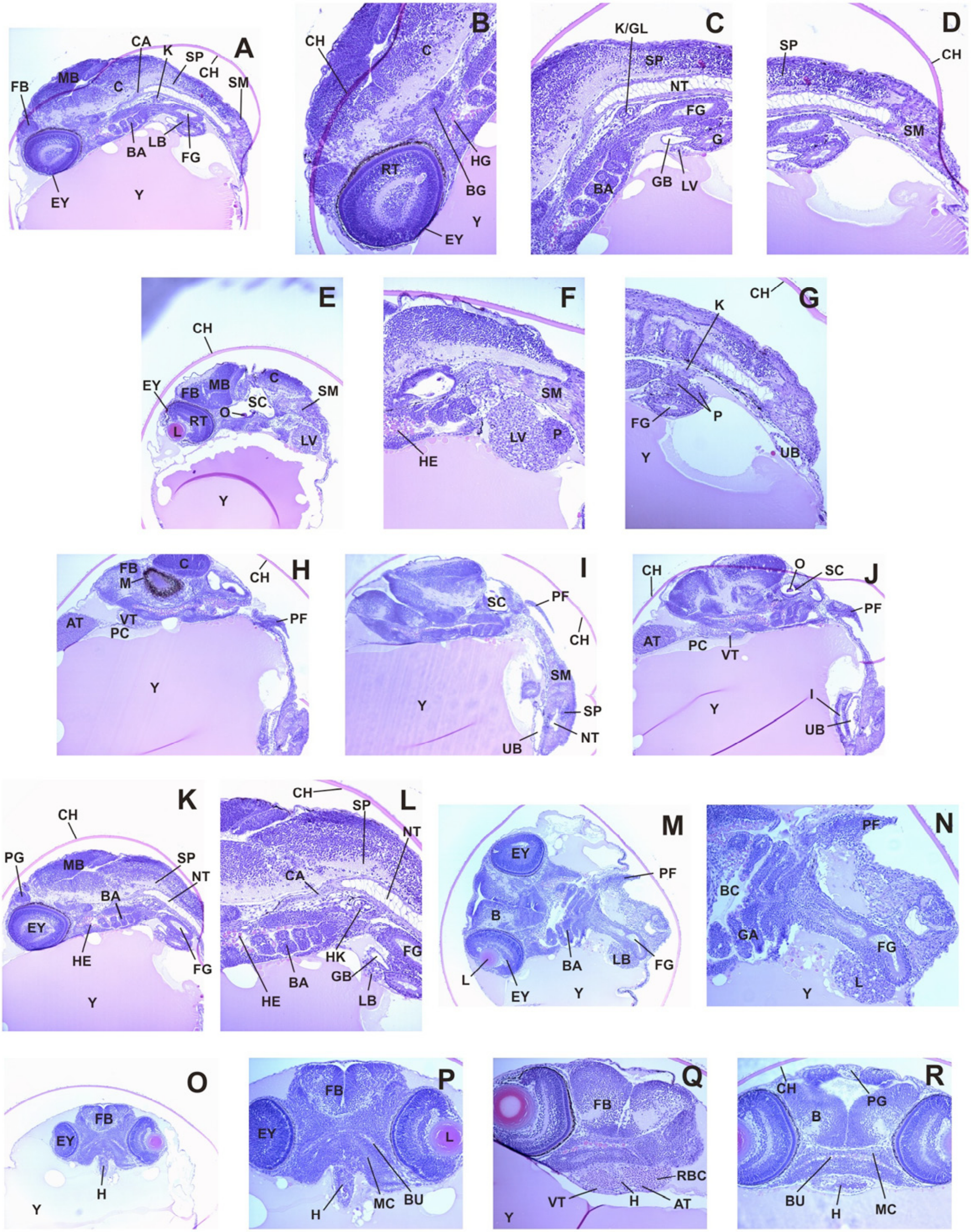
A
FB
MB
C
CA
K
SP
CH
SM
BA
LB
FG
EY
Y
B
CH
C
RT
HG
BG
Y
EY
C
K/GL
SP
NT
FG
G
GB
LV
BA
D
SP
CH
SM
E
CH
EY
FB
MB
C
SC
SM
O
RT
L
LV
Y
F
SM
LV
P
HE
G
CH
K
P
FG
Y
UB
H
FB
M
C
CH
AT
VT
PC
PF
Y
I
SC
PF
CH
SM
SP
NT
UB
Y
J
CH
O
SC
PF
AT
PC
VT
I
UB
Y
K
CH
PG
MB
SP
NT
BA
EY
HE
FG
Y
L
CH
SP
NT
CA
HK
HE
BA
GB
LB
FG
M
EY
PF
B
BA
LB
FG
L
EY
Y
N
PF
BC
GA
FG
L
Y
O
FB
EY
H
Y
P
FB
EY
L
H
MC
BU
Q
FB
RBC
VT
H
AT
Y
R
CH
PG
B
BU
H
MC

Additional File 2. *Fundulus heteroclitus* embryo histology at stage 31. AT-atrium; B-brain; BA-branchial arches; BC-branchial chamber; BG-basal ganglia; BU-buccal cavity; C-cerebellum; CA-cartilage; CH-chorion (displaced during processing/sectioning); EY-eye; FB-forebrain; FG-foregut; G-gut; GA-gill arches; GB-gall bladder; GL-glomeruli; H-heart; HE-unicellular glands containing hatching enzyme; HK-head kidney; I-intestine; K-kidney; L-optic lens; LB-liver bud; LV-liver; M-melanin; MB-midbrain; MC-mandibular cartilage; NT-notochord; O-ossicle; P-pancreas; PC-pericardial cavity; PF-pectoral fin; PG-pineal gland; RBC-red blood cells; RT-retina; SC-semi-circular canal; SM-skeletal muscle; SP-spinal cord; UB-urinary bladder; VT-ventricle; Y-yolk.

A-B. Near complete parasagittal section of the stage 31 *Fundulus* embryo (a-10X; b-20X). At the rostral-most extent of the embryo, the eye with lens and differentiating layers of retina are shown. Continuing with the central nervous system the rostral-most portion of the midbrain is the future optic tectum. Just caudal to this is the cerebellum continuing into the spinal cord, an elongated layer of cartilage is just ventral to the cerebellum and forms part of the cerebral vault. The basophilic structures immediately ventral to the cerebellum and rostral to the cartilage are apparent but not definitively identified. Candidate structures include: basal ganglia and/or pituitary. Between the aforementioned structures and the underlying yolk is a region of the branchial cavity including portions of four branchial arches. The eosinophilic single cells of the branchial and pharyngeal mucosa are remnants of hatching gland cells. Little to no differentiation of respiratory portions of the gill is seen at this time. Immediately caudal to the branchial cavity is the kidney showing sections through five individual tubules. Just caudal, appearing ventral, due to the curvature of long axis of embryo, is the liver. Two rounded structures between the liver and the adjacent foregut are bile ducts. The caudal-most portion of the embryo in this section reveals paraxial skeletal muscle.

C-D. Parasaggital sections of intestine, extramural glands, and longitudinal views of the spinal cord and notochord (20X). Dorsally (right side of the figure), brain and spinal cord descend toward the tail. Between the spinal cord and the structures of the kidney and intestine, there is a good depiction of the linear extent of a portion of the notochord. With respect to the kidney, the section through the glomerulus shows a portion of the vascular tuft. Other structures include a glancing section through the periphery of the liver bud and adjacent gallbladder, which is lined by a single layer of flattened epithelial cells. In the mesentery between the two profiles of the intestine, a pancreatic bud is shown.

E. Parasagittal section of a portion of the head and neck regions and the rostral- most portion of the abdominal cavity (10X). The lens of the eye, various layers of cells representing the differentiating retina and a portion of the anterior chamber of the eye are shown. Dorsal and somewhat caudal to the eye are a portion of the forebrain and the rostral-most portion of the midbrain. The triangular space caudal to the eye and forebrain is the semicircular canal. A small ossicle is shown within the space. Proceeding in a caudal direction, the dorsal-most structure is a portion of the cerebellum. Immediately beneath the cerebellum is a small cartilage. This is a portion of the future cranial vault. The rounded structure above the yolk sac is the liver bud. Between the liver bud and the caudal most portion of the brain is a region of skeletal muscle.

F. Liver (20X). The hepatocytes reveal a normal appearance with basophilic staining surrounding the nucleus and more peripheral regions of cells showing clear to pale staining, signifying glycogen deposits. There are numerous sinusoidal profiles whose lumens contain nucleated red blood cells. A cluster of basophilic cells is at the top of the field. Some of these have differentiated into definitive epithelial cells surrounding extrahepatic ducts.

G. Parasaggital section of Embryo mid-region (20X). The kidney contains portions of two glomeruli: the lower of which shows a thin walled circumferential epithelium of Bowman's capsule and a small region, with increased basophilia. This region is the urinary pole of the glomerulus. To the left of the glomeruli, the lumen of the branchial/buccal/pharyngeal region is

continuous with the lumen of the foregut. Epithelium within the former is arranged in multiple layers while that of the latter is a single layer with tall columnar epithelial cells. Due to the curving nature of the intestine, a transverse section and a longitudinal section are shown. Between the two portions of intestine are structures within the mesentery. The lower most portion resembles exocrine pancreas while the upper structure with numerous capillaries represents endocrine pancreas (*i.e.*, Islet of Langerhans).

H. Parasaggital section of heart chambers (10X). The atrium is shown at the uppermost left margin of the figure. Note the thin atrial wall. Moving to the right in contrast is the thick wall of the ventricle. The black material in the upper portion of the figure corresponds to melanin in the sclera of the eye.

I. Caudal end of the coelomic cavity (10X). The yolk sac is visible at the top of the field. The center of the field contains the distal end of the intestine. The urinary bladder is dorsal to the intestine and lies beneath the notochord and spinal cord.

J. Parasagital Secion, embryo midline (10X). As evidenced by the absence of the eye, the plane of this parasagittal section is much closer to the midline of the embryo. As a result, sections of the heart are visible. The atrium is shown at the extreme left margin and its lumen is nearly filled with nucleated red blood cells. Moving from left to right, the thicker walled ventricle is seen. Immediately above the atrium, material within the pericardial cavity is lightly eosinophilic and may indicate the presence of protein. Elements of the semicircular canals are shown immediately to the right of the brain, including the cells of the neuromast and a single ossicle. At the most dorsal margin of the figure is a section through a pectoral fin. Moving toward the caudal end of the embryo, we see a glancing section through the tail showing spinal cord and notochord. Immediately to the left of this we see the epithelium lining the urinary bladder and the mucosal epithelium associated with the distal end of the intestine.

K-L. Parasaggital sections (10X), structures found in the junction of pharyngeal and branchial cavities; the mid and hindbrain regions including extension into the spinal cord and notochord. Eye and the pineal gland dorsal most are seen. Ventral, midbrain structures lie immediately dorsal to the pharyngeal and branchial cavities. Within the branchial cavity, eosinophilic cells comprise the unicellular glands containing hatching enzyme (chorionase). Note portions of four branchial arches. Between the rostral most portion of the notochord and the roof of the branchial cavity is an elongated strip of cartilage. This will become a portion of the cranial vault. Examination of the foregut reveals two sections suggesting that the gut has undergone a curve from a truly midline longitudinal orientation to one that is somewhat transverse. A portion of the liver bud and adjacent gallbladder lie near the distal portion of foregut and between the two sections of foregut is the developing pancreas. In the tail section of the embryo, the spinal column is dorsal to the notochord as shown. The head kidney is in close proximity to the rostral-most notochord.

M. Horizontal-longitudinal section (10X). The left eye contains a lens and this organ is separated from the contralateral eye by the intervening forebrain. Caudal to the brain is the pharynx and associated branchial chamber containing a portion of four gill arches on each side. The light blue material surrounding the embryo proper is the yolk. A portion of the tail is at the top of the field, and a horizontal-longitudinal section through a portion of the foregut lies in the middle line between the tail and the aforementioned branchial cavity. The liver bud is lateral to the foregut on the left side of the developing embryo. The pectoral fin projects caudally on the right side.

N. Expanded partial view (20X). In the left of this field the gill arches are seen in the pharyngeal cavity. A horizontal-longitudinal section of the foregut extends caudally from the gill. The liver bud appears just below the terminal portion of the foregut and is surrounded by yolk material. The pectoral fin is at the top of the field.

O. Transverse section through the head at the level of the eyes (10X). The forebrain is between the left and right eye while the dorsal-most brain region is the midbrain. The ventral most structure in the mid line represents a portion of the heart and great vessels.

P. Transverse section of the atrioventricular passageway (20X). This section illustrates the atrioventricular opening. The left side of the embryo (right aspect of this figure) is cut more deeply (caudally) than is the contralateral side, which shows not only the eye, but also the lens within the eye. The overlying structures include: mandibular cartilage, the lumen of the buccal cavity, and elements of the forebrain between the eyes.

Q. Transverse section through rostral-most region of the head (20X). This section illustrates the relationship of the great vessels and heart to the overlying embryo. Between the bilateral eyes is the mucosa of the buccal cavity. The presence of mandibular cartilage is an indication that the plane of section is very close to the rostral tip of the organism. The heart (*i.e.*, the ventricle) is immediately ventral to the midline at the rostral portion of the head. Appearance of the forebrain can be used for orientation when viewing sagittal and parasagittal sections.

R. Transverse section at the rostral end of the head shows heart chambers and bilateral symmetry between the left and right eyes (20X). The red blood cells are grouped within a thin-walled atrium on the right, while the ventricle has a much more thickened wall and shows muscle bundles surrounded by endothelium within the chamber and fewer blood cells in the lumen.
