# Supplementary material for: Gene expression throughout a vertebrate's embryogenesis
Source: BMC Genomics. 2011 Feb 28;12:132. doi: 10.1186/1471-2164-12-132 (PMC3062618; doi:10.1186/1471-2164-12-132)
Supplement: Additional file 4 — F. heteroclitus stage descriptions. [file 1471-2164-12-132-S4.PDF]

Additional File 4. *F. heteroclitus* stage descriptions*.

STAGE (hr)	STAGE DESCRIPTION
**1** (0)	**Mature Unfertilized egg.** The mature spherical ovum is on average 2 mm in diameter, although the size varies and correlates to the size of the female, with larger females having larger but not necessarily more eggs. The egg is enveloped by the chorion – a thick 2-layer membrane consisting of inner protein fibrils and outer homogenous protein fibers. The micropyle looks like a small funnel-shaped indentation on the outer layer of the chorion's animal (uppermost) pole. At the center of the egg is the homogenous and opaque yolk covered with the small round cortical granules (yolk platelets) and oil droplets, which vary in size.
**2** (1.5 ± 0.25)	**1-cell stage.** Upon fertilization, formation of pre-vitalline membrane causes the egg to shrink away from the inner chorion membrane and the cortical granules disappear. The cytoplasm migrates to the micopyle (animal) pole of the egg and eventually forms an elevated blastodisc. Oil droplets migrate towards and eventually aggregate at the vegetal pole.
**3** (2.5 ± 0.20)	**2-cell stage.** The blastodisc undergoes first cleavage resulting in two blastomeres (cells) equal in size.
**4** (3.0 ± 0.44)	**4-cell stage.** The two blastomeres flatten and the second cleavage takes place, usually at the right angle of the first cleavage plane, forming 4 blastomeres of equal size.
**5** (5 ± 0.51)	**8-cell stage.** The third cleavage, which is parallel to the first, divides the four blastomeres into 2 parallel rows of four, resulting in 8 blastomeres. The blastoderm elongates along the 2nd cleavage axis, and arrangement of cells can sometimes appear slightly irregular.
**6** (6 ± 0.50)	**16-cell stage.** The fourth cleavage plane, which is parallel to the second, forms 4 parallel rows of 4 cells whose arrangement can also appear irregular. By the end of this stage, most of the oil droplets migrate towards the vegetal pole.
**7** (7.5 ± 0.50)	**32-cell stage.** The fifth cleavage plane divides 16 outer cells meridionally, while the inner 4 cells are divided horizontally, creating an inner and outer layer of cells. Later in this stage the outer cells undergo rearrangement and appear irregular with respect to each other.
**8** (8.5 ± 0.51)	**Early Morula (64-128 cells).** The sixth cleavage, which is difficult to follow, results in the 64 smaller cells, with no apparent increase in blastodisc size. The cells are clustered together forming 2-3 layers in the center.
**9** (9.5 ± 0.51)	**Late Morula (256-512 cells).** Depending on their position within a blastoderm, the cells have different cleavage planes and undergo further divisions. The peripheral cells flatten, and the overall cell arrangement results in the formation of 3-4 layers.
**10** (10 ± 0.70)	**Early Blastula.** The ongoing divisions result in smaller and more numerous cells, with central cells being smaller than the outer. There is no apparent change in blastoderm size, which appears thick. The nuclei of cells from the outer layer migrate out, and form a few rows in the yolk syncytial layer known as periblast.
**11** (12 ± 1.32)	**Flat Blastula.** The blastoderm sinks, flattens, and caps the yolk. The individual cells cannot be resolved and the blastoderm appears as a homogenous mass.
**12** (15 ± 1.83)	**Pre-early gastrula.** The circular blastoderm expands further over the yolk. The cell layers appear thicker on one side, as the outer layer of cells migrates towards the vegetal pole. The blastoderm appears as a biconvex disc with the upper layer being more convex. Under higher magnification, 4-5 bands of irregular migrating nuclei can be observed at the margin of a blastodisc.
**13** (19 ± 1.63)	**Early gastrula.** The blastoderm flattens and expands further to cover less than 20% of the yolk mass. The band of cells on one side of the periblast continues to thicken, forming a dorsal lip of the blastoderm. The fine droplets concentrate at the animal pole, the site where the blastopore will eventually close.
**14** (21 ± 1.25)	The blastoderm expands further to cover about 25% of the yolk's surface. The peripheral cell layers of the dorsal lip thicken further, marking the future location of the embryonic shield.
**15** (25 ± 2.38)	**Pre-mid-gastrula.** The blastoderm extends further over about 30% of the yolk, a process termed epiboly, causing the relative thinning of this layer, while the embryonic shield continues to build up increasing size around the dorsal lip.
**16** (28.5 ± 2.24)	**Pre-mid-gastrula.** The blastoderm covers more than 33% of the yolk sphere. The dorsal lip elongates and the subsequent cell divisions cause further build up of the embryonic shield, which is

	greater at one portion of the blastoderm.
**17**<br>(31 ± 1.75)	**Mid-gastrula.** The blastoderm covers 50% of the yolk, and the streak caused by thickening of the embryonic shield, known as the embryonic axis, is apparent.
**18**<br>(34 ± 1.75)	**Late Gastrula.** The blastoderm covers 75% of the yolk and the embryonic shield elongates and becomes more pronounced, appearing as a narrow streak. The fine droplets concentrate further at the vegetal pole.
**19**<br>(38 ± 2.51)	**Early Neurula.** The blastoderm covers most of the yolk, with the small area at the vegetal pole, the blastosphere, exposed. The embryonic axis (neural keel or neurula) broadens, with the distinctive thickening anteriorly, forming a head (cephalic) region. The bilateral thickening of the head region indicates the presence of rudimentary optic vesicles. Small vacuoles (Kupffer's vesicles) are present at the caudal (posterior) end, which is in contact with the small blastopore.
**20**<br>(42 ± 3.42)	**Late Neurula.** The blastopore is closed and the blastoderm covers the entire yolk. The upper cephalic region widens and forms two optic buds. The further thickening of the head region results in formation of the brain, forebrain, midbrain, and hindbrain regions. A group of mesodermal cells aggregate ventrally to the embryonic axis, marking the location of the future anterior somite region.
**21**<br>(44 ± 4.67)	**3-4 somites.** The embryo increases in size and further mesodermal segmentation forms of 3-4 somites apparent bilaterally in the embryo's midline. The optic lobes in the head region are more defined.
**22**<br>(49 ± 4.42)	**6-9 somites.** The brain regions are well defined and the optic cup is formed within the optic lobe. The number of somites increases. The small strands on the yolk's surface indicate the initiation of the blood islands. Outlines of the pericardial cavity are located anterior to the head. The two otic placodes (auditory vesicles) are caudal in the cephalic region. The Kupffer's vesicle is located anterior to the tail region, and the fine droplets aggregate under the tip of the tail.
**23**<br>(54 ± 5.39)	**Heart formation.** The embryo grows in size and the main structures differentiate further with 10-12 somites now apparent. The main brain region is well defined. The optic vesicles differentiate further and the optic lenses are formed, but do not protrude out of the optic cup. The outline of the pericardial cavity can be viewed bilaterally to the mid and hindbrain. The flat body cavity forms on the yolk's surface bilateral to the mid and hindbrain. The heart anlagen (primitive tubular heart) is under the head, caudal to the mid-brain and anterior to the hindbrain. The blood islands grow in size, and small pigment cells appear scattered over the yolk sac. The tail region is round and attached to the yolk.
**24**<br>(65 ± 5.32)	**Heart beat initiation.** The embryo occupies almost 50% of the yolk's surface and there are 14-16 somites. The body cavity extends towards the caudal end of the eye vesicles, and the otocysts (differentiated otic vesicles) become more prominent. The optic lenses are spherical, filling in the optic cups. The outlines of Cuvierian ducts appear laterally around the yolk sac, but are incomplete. The anterior heart region is under the forebrain and eye vesicles. The elongated heart pulsates slowly (30-40 beats per minute or bpm) from the venous to the arterial end, while the melanophores appear larger on the yolk surface and the larger blood islands form a linkage. Circulation is not observed and the tail region remains attached to the yolk, having not extended in the caudal direction.
**25**<br>(72 ± 5.22)	**Onset of circulation.** The heart rate increases (>50 bpm) and the pericardial cavity extends, separating the head from the underlying yolk. The concentrated blood islands form at the venous end of the heart, and the tip of the tail is slowly pushed out of the blood islands resulting in a slow and pulsating circulation. Later in this stage, the heart rate increases (>60 bpm), and the circulation stabilizes, with the blood cells moving through the three vitelline veins. The ducts of Cuvier extend further laterally around the yolk. The tip of the tail, with the vitello-caudal vein in the view, detaches from the yolk. The optic lenses have increased in density due to an increase in melanin pigmentation. The embryo has 24 somites.
**26**<br>(80 ± 5.71)	The heart elongates and curves at the cranial end. The blood can be seen circulating anteriorly to the hindbrain. The eyes are darker, while the paired otoliths appear as dark, round granules. The embryo contraction is first apparent in musculature of the few cranial somites. The vitelline caudal vein extends from the tip of the elongated tail onto the yolk sac, branching further into the vitelline vessels. There are 30 somites.
**27**	Embryo contractions, in the anterior somites, become more pronounced and more frequent.

(90 ± 7.80)	Volume of the yolk gradually decreases. The embryo occupies about 2/3 of the yolk sphere. The otoliths look like small granules within the larger otocyst. The rudiments of pectoral fins protrude as buds posteriorly to the base of the ducts of Cuvier. The heart rate is >80 bpm, and the ventricles form an irregular heart chamber. The body cavity expands, with the gut attached to the yolk sac. The melanophores expand on the yolk's surface and the tail bends laterally in some embryos. A total of 34 somites are formed at this stage.
**28** (102 ± 11.35)	The heart rate is 80-100 bpm, and the arterial end of the heart shifts slightly to the right. The eyes appear darker due to apparent thickening and melanin pigmentation. The body cavity extends further, lifting the gut off the yolk sac, with the blood vessels along the gut now in view. The liver rudiment appears on the left of the first pair of somites, and the round, dark gall bladder is seen to the left of the midline anterior to the liver which is still attached to the yolk mass. A small, inactive urinary bladder appears as a bilobed structure at the caudal end, which is still attached to the yolk. There are about 35 somites formed.
**29** (110 ± 13.31)	The embryo is wrapped three-fourths of the way around the remaining yolk. The heart is further repositioned, with the slight bend at the rostral end to the left and the arterial end to the right. In lateral view, the atrium and ventricle are visible under the head. The overall size of the eyes and their pigmentation increases. The pectoral fins extend further from the trunk, while the liver rudiment enlarges in left view at somites 3-4. The urinary bladder appears as a small bilobed organ above the pectoral fin.
**30** (120 ± 13.31)	The embryo is wrapped five-sixths of the way around the remaining yolk, with all of the structures further differentiated. The heart rate is at about 100-120 bpm. The retina is darker due to pigment accumulation and cellular growth, and the pectoral fin stretches slightly over the lateral line. The gallbladder increases in size and assumes a greenish color. The Ducts of Cuvier move towards the surface of the yolk sac, and the circulation can be observed throughout the liver with the hepatic vein draining into the left Duct of Cuvier. The urinary bladder differentiates further into a small bilobed structure but does not form a precipitate. The tail straightens out completely and moves frequently, reaching above the hindbrain during lateral flexion. Melanophores increase further in size. The embryo's surface pigmentation increases, but is localized to spots on the surface.
**31** (140 ± 12.06)	The heart rate reaches 120-130 bpm (the average heart rate among 10 families of 3 embryos/family was 123.3 ± 1.09 bpm), and the heart chambers are differentiated under the cephalic region into two distinct chambers – the atrium on the left, and the ventricle rostrally. The blood is pushed out from the atrium to the ventricle although the ventricle does not fully fill out. Caudal to the heart, the vein splits into the left and right cardinal veins, which join posteriorly, forming the common cardinal vein and eventually emptying into the heart's sinus venosus. The blood circulates through a closed channel system and flows to each side of the head *via* the carotid artery. The dorsal aorta in the trunk continues as a caudal artery as it enters the tail and subsequently bifurcates dorsally and ventrally to enter the caudal fin. A faint circulation in the vessel parallel to the pectoral fin is apparent. The liver is larger, and the circulation through the sinusoids can be observed. The bilobed urinary bladder can now be seen to contain precipitate.
**32** (160 ± 11.72)	The head elongates and gradually "straightens out". The retina is darker, and the outline of the lens can be depicted with increased transillumination. The operculum outline is visible anterior to the otocysts. The aorta increases in size and heartbeat is stronger with increased volume being pushed through the chambers. The liver and gall bladder are closer when observed laterally, and the hepatic vessel blood flow is pronounced, with the blood draining into the left Duct of Cuvier. The tip of the tail can reach the eyes and its movements are frequent. A single vessel forms within the membranous pectoral fin, and the vessels begin to radiate within the caudal fin.
**33** (180 ± 10.31)	The yolk sac is further reduced and the embryo exhibits frequent movement. The retina is larger and heavily pigmented. The head extends out farther in a rostral direction, separating the embryo from the yolk surface anteriorly at the level of the hindbrain. The mouth forms under the head and rudimentary lower and upper jaws are seen; the lower jaw is firmly attached to the outer surface of the adjacent yolk. The membranous pectoral fin movement is weak and irregular, and several melanophores can be observed along the faint rays of the caudal fin.
**34** (195 ± 9.46)	Two small olfactory pits appear on the frontal bone between the eyes, and the oral cavity is composed of the upper and the lower jaw. The latter remains loosely attached to the yolk surface. Later in this stage, the lower jaw separates from the yolk, and the mouth opens frequently. The

	hatching enzymes concentrate in the buccal epithelium, ventral and somewhat caudal to the eyes. The gill arches are apparent but poorly differentiated, and the heart rate is stabilized, remaining between 120-130 bpm. A small vacuolar body between the pectoral fins outlines the formation of the future swim bladder. The tail can reach the middle of the eyes and the ventral fin is formed along the tail. Blood circulates throughout the vessels radiating through the caudal fin, and the embryo's movement is frequent, accompanied by an uncoordinated fluttering of the pectoral fins.
**35**<br>(212 ± 12.80)	**Pre-Hatching.** Continuous elongation of the head causes further separation of the ventral head region from the yolk. The eyes are large and move frequently. The liver increases in size and obscures the view of the gall bladder. Operculum (gill cover) margins are now well defined. The tail can reach beyond the caudal border of the eye. The heart rate remains stable between 120-135 bpm (the average heart rate among 10 families of 3 embryos/family was 125.3 ± 0.64 bpm). The contraction of the embryo, resembling swimming movements, is accompanied by frequent but unsynchronized fluttering of the pectoral fins. The tip of the tail can reach beyond the otic vesicle and the embryo often rotates within the chorion. A strong tail movement often results in the complete rotation of the embryo.
**36**<br>(226 ± 11.25)	**Hatching.** The more frequent opening of the lower jaw indicates the beginning of the hatching process, during which the hatching enzyme is released from unicellular glands in mucosa of buccal and pharyngeal regions. The chorion eventually lyses and the embryo breaks free tail-first. The tail straightens immediately upon hatching, and the embryo is able to assume swimming movements within the hatching medium.
**37**<br>(238 ± 10.01)	The yolk is significantly reduced in size and now extends from under the pectoral fins to the anus, which is located approximately at the body's midline. The overall embryo movements are frequent and well coordinated, with pectoral fins fluttering continuously. The early formation of dorsal and ventral fins contributes to the maturation of the motor skills. The lower jaw and the opercular movements appear more synchronized, and the motor activity becomes more efficient overall.
**38**<br>(256 ± 9.95)	Viewed laterally, the yolk sac covers the anterior region of the operculum and extends pass the caudal end of the pectoral fin. The swim bladder increases in size, while the dorsal and ventral fins become more prominent. The rays within the pectoral and caudal fins are now well defined. The embryo's movement improves, but the movements between the operculum and the pectoral fins are not completely synchronized.
**39**<br>(278 ± 14.78)	The diminishing yolk sac covers the area beneath the pectoral fins and the embryo swims frequently and efficiently.
**40**<br>(290-374<br>± 30.29)	The yolk is completely absorbed, marking the initiation of the larval stage. The embryo movement is characterized by synchronized activity of the operculum and constant movement of the pectoral fins. The volume of the swim bladder is sufficient to enable the larva to be suspended within the water column.

*Average times to stage in hours (hr) are in parentheses ± standard deviations from 10 families of 3 embryos each.
