# Supplementary material for: Gene expression throughout a vertebrate's embryogenesis
Source: BMC Genomics. 2011 Feb 28;12:132. doi: 10.1186/1471-2164-12-132 (PMC3062618; doi:10.1186/1471-2164-12-132)
Supplement: Additional file 8 — Representative pre- and post-normalization MA plots. [file 1471-2164-12-132-S8.PDF]

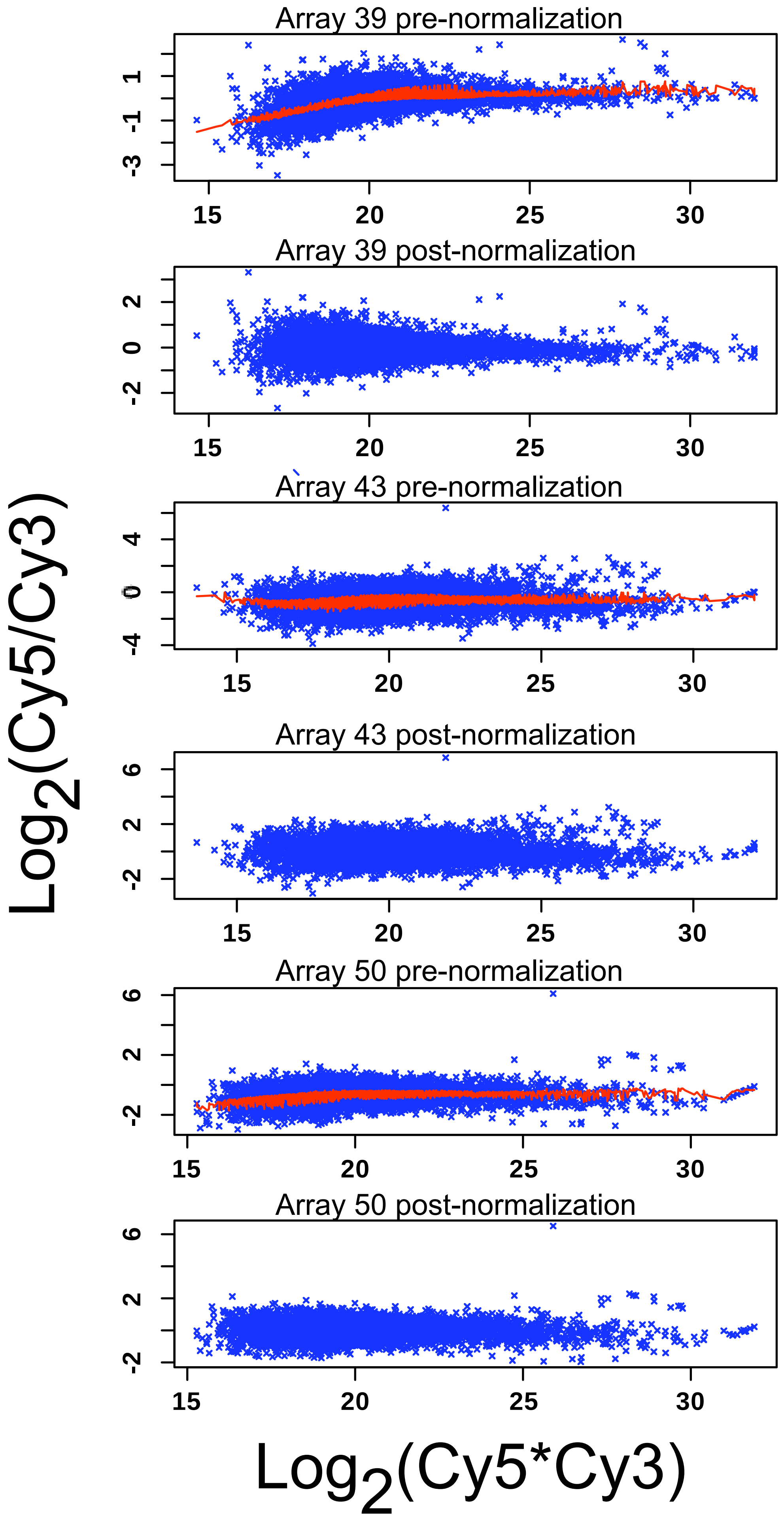

Additional File 8. Representative pre- and post-normalization MA plots. Normalized Cy5 and Cy3 intensity values are plotted as $log_2(Cy5/Cy3)$ versus $log_2(Cy5*Cy3)$.
